# Utilizing G2/M retention effect to enhance tumor accumulation of active targeting nanoparticles

**DOI:** 10.1038/srep27669

**Published:** 2016-06-08

**Authors:** Guanlian Hu, Xingli Cun, Shaobo Ruan, Kairong Shi, Yang Wang, Qifang Kuang, Chuan Hu, Wei Xiao, Qin He, Huile Gao

**Affiliations:** 1 Key Laboratory of Drug Targeting and Drug Delivery Systems, West China School of Pharmacy, Sichuan University, No. 17, Block 3, Southern Renmin Road, Chengdu 610041, China

## Abstract

In recent years, active targeting strategies by ligand modification have emerged to enhance tumor accumulation of NP, but their clinical application was strictly restricted due to the complex preparation procedures, poor stability and serious toxicity. An effective and clinical translational strategy is required to satisfy the current problems. Interestingly, the internalization of NP is intimately related with cell cycle and the expression of receptors is not only related with cancer types but also cell cycle progression. So the cellular uptake of ligand modified NP may be related with cell cycle. However, few investigations were reported about the relationship between cell cycle and the internalization of ligand modified NP. Herein, cellular uptake of folic acid (FA) modified NP after utilizing chemotherapeutic to retain the tumor cells in G2/M phase was studied and a novel strategy was designed to enhance the active targeting effect. In our study, docetaxel (DTX) notably synchronized cells in G2/M phase and pretreatment with DTX highly improved *in vitro* and *in vivo* tumor cell targeting effect of FA decorated NP (FANP). Since FA was a most common used tumor active targeting ligand, we believe that this strategy possesses broader prospects in clinical application for its simplicity and effectiveness.

In the past decades, chemotherapy was one of the major methods for the treatment of cancer^1^,^2^. However, the application of chemotherapy was hindered by low anti-tumor efficacy and systemic toxicity due to their nonspecific distribution^3^,^4^. Nanomedicines were considered as prospective formulations to improve the target ability and elevate the accumulation of chemical medicines in tumor sites^5^,^6^, and several nanomedicines have been approved, such as Doxil and Abraxane. To further improve the tumor accumulation, various approaches have been applied, including ligand modifying^7^,^8^,^9^, size changing^10^,^11^ and surface charge changing^12^,^13^, which have already been proved to facilitate cellular uptake and improve anti-tumor efficacy in animal experiments.

Besides, recent studies have proved that cell cycle has an effect on the cellular internalization^14^. Cellular uptake of nanoparticles (NP) in different stages of cell division showed a significant difference^15^. One integral cell cycle progression is composed of G0, G1, S, G2 and M phase. The cellular uptake ability of each phase of cell cycle was ranked as below: G2/M > S > G0/G1^14^,^16^. Many chemotherapeutics, such as paclitaxel, cabazitaxel and docetaxel (DTX), could arrest the tumor cells in G2/M phase^17^. Consequently, these G2/M arresting agents could significantly improve the tumor cell internalization of NP^18^.

What’s more, the expression of receptors was not only related with the cancer types but also cell cycle progression^19^,^20^, leading to different response to targeted NP during different cellular cycle progression. For instance, peripheral benzodiazepine receptor was cell cycle-related expression in human breast cancer cell lines^21^. However, current studies didn’t investigate the internalization of targeted NP in different cell cycle phases. Though, our previous study has proved that pretreatment with DTX could effectively arrest cells in G2/M phase, leading to enhanced cellular uptake of NP, it is not clear whether arrest cell cycle in G2/M phase could increase the tumor cell internalization of ligands modified active targeting NP. As a widely used strategy, many targeting ligands were applied to enhance the specificity and tumor targeting ability of NP, and several targeted nanomedicines have entered human clinical trials and preclinical pipeline^22^,^23^,^24^. Among them, folic acid (FA) is a widely used tumor targeting ligand^25^. Folic acid receptor (FR) is commonly overexpressed in many human cancer cells and has high affinity with FA^26^,^27^,^28^. On the contrary, the expression of FR on normal tissues and cells is low^29^.Therefore FA conjugated nanomedicines could actively target to tumor^30^. Some of FA conjugated formulations are now under clinical evaluation, for instance, EC0225 represents the first FA-drug conjugation to be evaluated in clinical trials^31^. Thus, in this study, FA was employed to functionalize the NP for active tumor targeting, and the cellular uptake of FA conjugated PEG-PCL NP (FANP) during different cell cycles was studied.

As previously reported by our lab, DTX is a first line chemotherapeutic that can arrest cells in G2/M phase and DTX pretreatment could indeed improve tumor targeting and cell internalization of NP^18^. Thus in this study, we would combine the G2/M phase retention effect of DTX and tumor active targeting effect of FANP to evaluate whether this novel strategy could further improve the tumor targeting delivery. The cellular uptake on FR positive A549 cells and negative L929 cells of FANP after pretreated with DTX was studied. Furthermore, the cellular uptake on DTX pretreated cells of FA modified liposome (FA-LIP) was set as control to clarify this universal mechanism. *In vivo* fluorescence imaging, *ex vivo* fluorescence imaging and tumor slices was used to evaluate the distribution of different NP with or without pretreatment with DTX. This study may open up the possibility of increasing targeting delivery efficacy through simply combining active targeting nanomedicines with clinical available G2/M phase retained chemotherapeutics and provided appeal for the development of novel therapeutic strategies by application of the existing formulations in clinic.

## Results

### Synthesis of FA-PEG-PCL

FA-PEG-PCL copolymers were synthesized through the reaction between the amino groups of FA-EDA and the carboxylic groups of PEG-PCL. Supplementary Fig. S1 showed the spectra of EDA-Boc, FA-EDA-Boc, FA-EDA, PEG-PCL and FA-PEG-PCL. The representative peaks of FA at 6.4–8.8 ppm and the representative peaks of EDA-Boc at 1.4, 2.4, 3.2–3.8 ppm was found in ^1^H-NMR spectra (Dimethyl Sulfoxide-D6) of FA-EDA-Boc, indicating FA-EDA-Boc was successfully synthesized. After FA-EDA-Boc was reacted with TFA, FA-EDA was synthesized. The representative peak of Boc at 1.4 ppm in ^1^H-NMR spectra (Dimethyl Sulfoxide-D6) of FA-EDA was disappeared, and the molecular weight of FA-EDA determined by Mass spectra (479.2) was consistent with the theoretical value of 479.4 (Supplementary Fig. S2), indicating FA-EDA was successfully synthesized. The ^1^H-NMR (CDCl_3_) spectra of FA-PEG-PCL was consistent with the structure of the expected polymers^32^, signals at 3.4–3.6 ppm were assigned to methylene protons of the PEG backbone, the signals at 4.06 ppm and 2.2 ppm belong to methylene group of the caprolactone unit, the signals at 2.00–2.40 ppm and the resonance at 4.50 ppm were attributed to the methylene in the R-position for the amide groups of FA, the signals at 6.61 ppm and the resonance at 8.66 ppm were attributed to the protons of the aromatic ring of FA. In summary, FA-PEG-PCL was successively synthesized.

### Characterization of NP

**Figure 1:**
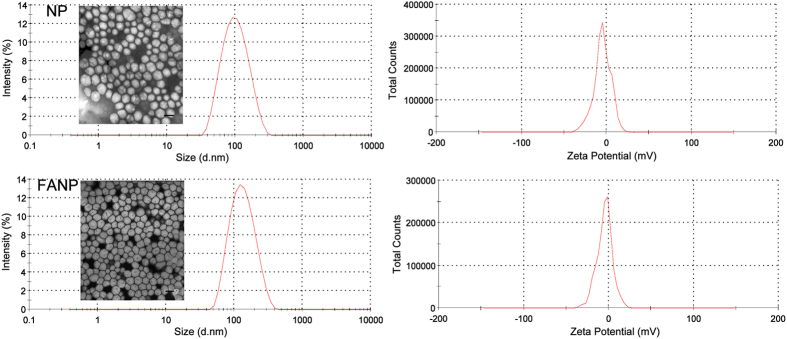
The particle sizes and zeta potentials of NP and FANP via DLS and TEM, scale bar represents 100 nm.

The average diameters of NP and FANP were 91.45 nm and 119.7 nm respectively (Fig. 1, Supplementary Table S1). FANP displayed a small increase in size maintaining a narrow size distribution as demonstrated by dynamic light scattering (DLS) measurements. TEM images demonstrated uniformly spherical structure of NP and FANP. In addition, both NP and FANP presented a little negative zeta potentials under similar conditions. Zeta potentials of NP and FANP was −4.65 mV and −4.82 mV. According to Supplementary Table S2, the average sizes of PEGylated liposome (LIP) and FA-LIP were 103.0 nm and 101.7 nm. These liposomes displayed similar negative zeta potentials with NP and FANP.

### Cellular uptake after DTX pretreatment

**Figure 2:**
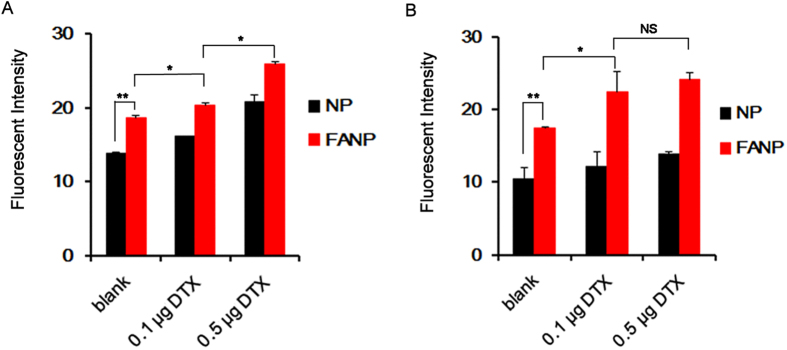
The cellular uptake after pretreatment with 0.1 μg or 0.5 μg DTX for 24 h on A549 cells (**A**) and U87 cells (**B**). *, ** and *** represent *P* < 0.05, *P* < 0.01 and *P* < 0.001 respectively between the marked groups and N.S indicated no significant difference.

Cellular uptake of FANP was significantly higher than NP on A549 and U87 cells. On the other hand, cellular uptake of NP and FANP also measured on FR negative L929 cells. No significant increase was observed compared FANP to NP (Supplementary Fig. S3), confirming FANP could bind to FR on cell membrane and lead to enhanced cellular uptake by endocytosis^33^. As demonstrated previously, low dosage of DTX arrested more cells in G2/M phase^18^, thus we evaluated the influence of DTX on cellular uptake of NP and FANP (Fig. 2). The cellular uptake after pretreated with DTX was obviously enhanced in comparison with the control group and showed positive correlation with the concentration of DTX on A549 cells and U87 cells, implying that DTX could facilitate cellular internalization of NP. More importantly, the cellular uptake of FANP increased after pretreated with DTX, displaying the superiority of combinatorial therapy between active tumor targeting and DTX pretreatment. To clarify the universal mechanism, we also evaluated the cellular uptake of LIP and FA-LIP (Supplementary Figs S4 and 5). Cellular uptake of LIP and FA-LIP displayed no significance on L929 cells while cellular uptake of FA-LIP was significantly higher than LIP on A549 cells. After pretreated with 0.1 μg/mL DTX, cellular uptake of FA-LIP on A549 cells was also increased 2.11 times than untreated group. Therefore, combining DTX pretreatment with FANP might be a promising strategy for enhancing tumor targeting drug delivery.

### Cell cycle synchronization

**Figure 3:**
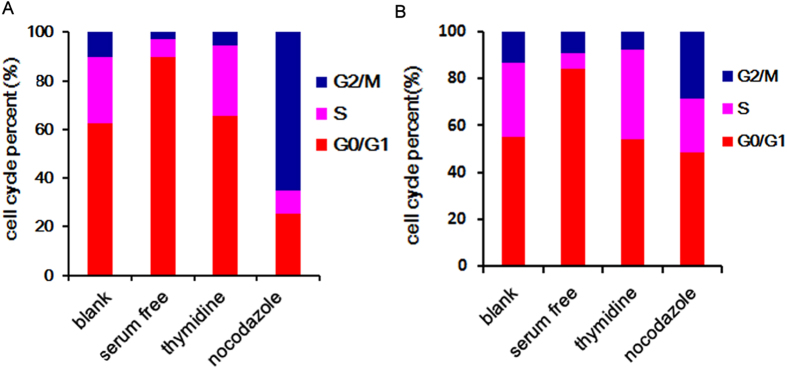
The distribution of cell cycle with drug-synchronized A549 cells (**A**) and U87 cells (**B**) after different treatment (serum free, G0/G1 phase; thymidine, S phase; nocodazole, G2/M phase).

Normal cell cycle consists of four phases: G0/G1, S, G2 and M, which could be determined by analysis the nuclear DNA content through PI staining^34^. In our study, classic method was adopted to synchronize the cells in different cycle. Histogram of DNA fluorescence can be observed to show the proportion of A549 cells in G0/G1 (normal DNA content, 2N), S (DNA synthesis) and G2/M (double DNA content, 4N) phases. Cell cycle analysis displayed that >80% A549 cells were arrested in G0/G1 after serum starvation for 48 h (Supplementary Fig. S6B and Fig. 3A). Treatment with 1 mmol/L thymidine for 16 h synchronized >30% of the A549 cells in S detected by cell cycle test (Supplementary Fig. S6C and Fig. 3A). A549 cells in G2/M phase were significantly increased from 10.1% to 65.1% compared with asynchronous cells (Supplementary Fig. S6D and Fig. 3A). The similar trend was also observed in U87 cells (Supplementary Fig. S6F–H and Fig. 3B). All the data confirmed that a large number of cells were synchronized in one phase by special method. Thus the synchronous cells could be used for evaluation of cellular uptake of NP and FANP in different cell phases.

### Cellular uptake after cell cycle synchronization

**Figure 4:**
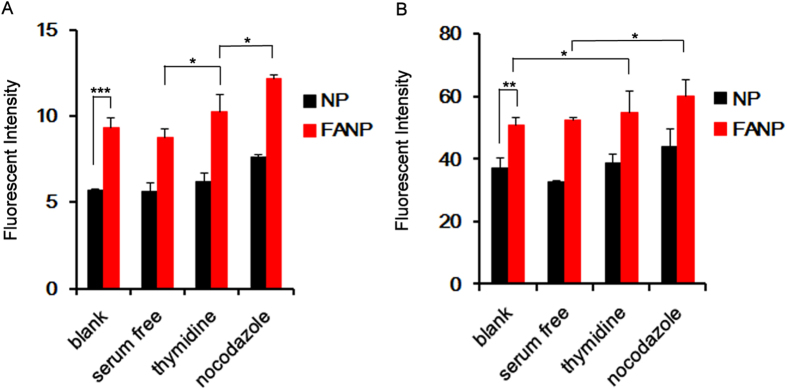
Cellular uptake of drug-synchronized A549 cells (**A**) and U87 cells (**B**) after different treatment (serum free, G0/G1 phase; thymidine, S phase; nocodazole, G2/M phase). *, ** and *** represent *P* < 0.05, *P* < 0.01 and *P* < 0.001 respectively between the marked groups.

Based on the previous method of cell cycle synchronization, the cellular uptake of NP and FANP was further evaluated in different cell cycle progression. The results were extremely in accordance with previous studies^14^,^15^,^18^. According to Fig. 4, the uptake of FANP in all phases of both A549 cells and U87 cells was higher than NP, demonstrating the FA modification could improve tumor cell internalization. Furthermore, the cellular uptake of FANP was ranked as follows in both cells: G2/M > S > G0/G1, which proved that the G2/M retention could be used for improving tumor cell internalization of active targeting NP.

### *In vivo* imaging

**Figure 5:**
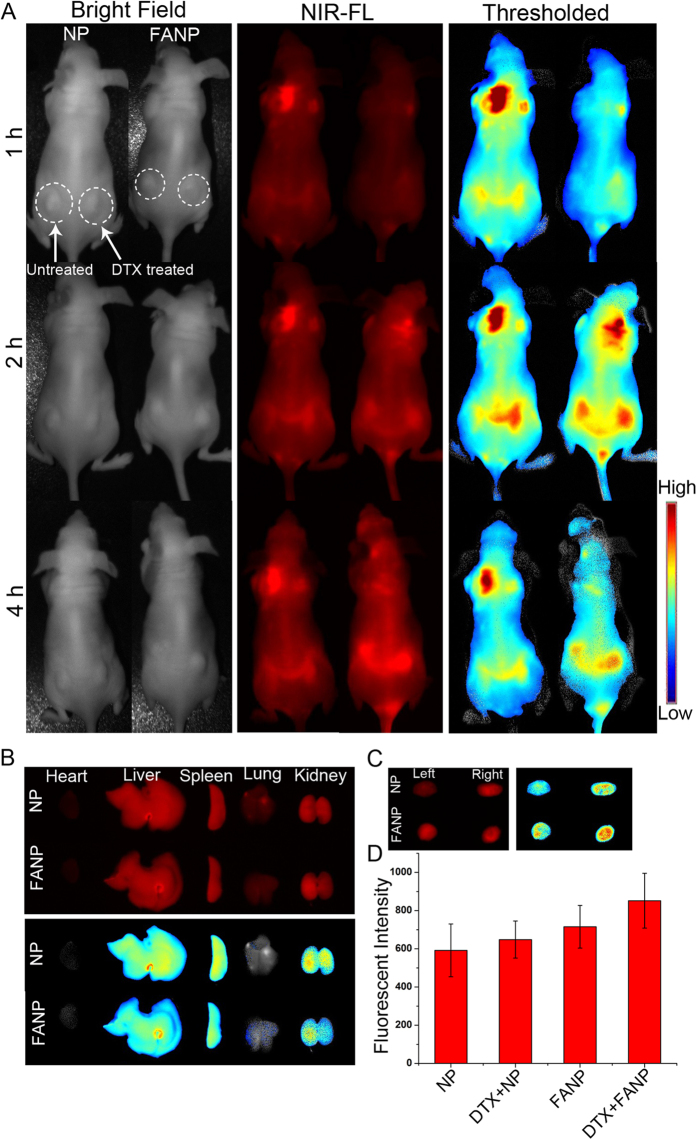
(**A**) *In vivo* fluorescence imaging of the A549 tumor-bearing nude mice at 1, 2 and 4 h after injection of different NP with or without pretreatment with DTX. (**B**) *Ex vivo* fluorescence imaging of normal tissues of A549 tumor-bearing nude mice after mice were sacrificed at 4 h post-injection. (**C**) *Ex vivo* fluorescence imaging of tumor of A549 tumor-bearing nude mice after mice were sacrificed at 4 h post-injection. NP represented mice were administered with DiR loaded NP; FANP represented mice were administered with DiR loaded FANP. The right tumors of the A549 tumor-bearing nude mice were pretreated with DTX before administration of DiR loaded NP or FANP. The left tumors were pretreated with the solvent of DTX instead. (**D**) Semi-quantitative analysis of fluorescent signals from tumors after 4 h injection with different particles. DTX + NP represented tumor pretreated with DTX before administration with DiR loaded NP; DTX + FANP represented tumor pretreated with DTX before administration with DiR loaded FANP; NP represented mice were only administered with DiR loaded NP and tumor hadn’t been pretreated with DTX; FANP represented mice were only administered with DiR loaded FANP and tumor hadn’t been pretreated with DTX.

The potential use of this novel strategy was further investigated *in vivo*. We initially selected A549 tumor-bearing nude mice as animal model. The mice were introduced with A549 xenografts on both sides of flank and the right one were pre-injected with DTX for 24 h to arrest tumor cells in G2/M. Subsequently the mice were intravenous injected with DiR loaded NP or FANP. The fluorescence in DTX-pretreated tumors was significantly higher than that of non-treated tumors (Fig. 5A), confirming that DTX pretreatment could elevate the tumor accumulation of NP, which was consistent with previous report^18^. More importantly, the tumor accumulation of FANP in DTX treated tumor was higher than untreated tumor on the same mouse, suggesting DTX pretreatment was useful in improving the tumor targeting effect of ligand modified NP. The results demonstrated combining DTX pretreatment with targeting ligand modification was useful not only *in vitro* but also *in vivo*.

### *Ex vivo* fluorescence imaging

The mice were sacrificed at 4 h post-injection and the dissected organs were imaged. According to semi-quantitative analysis of fluorescent signals from tumors after 4 h injection with different particles in Fig. 5D, pretreatment with DTX largely enhanced the accumulation of NP in tumor sites, and FANP + DTX group showed the best tumor targeting effect than NP in both DTX pretreated mice and untreated mice. The fluorescent intensity in FANP + DTX group was 1.2 fold higher than FANP group, which was consistent with the *in vivo* fluorescence imaging in Fig. 5A and *ex vivo* fluorescence imaging in Fig. 5C. The distribution in other tissues was also investigated (Fig. 5B and Supplementary Fig. S3). The fluorescence mainly focused in liver and spleen, which were the main organs to eliminate foreign materials.

### The distribution in tumor slices and normal tissue slices

**Figure 6:**
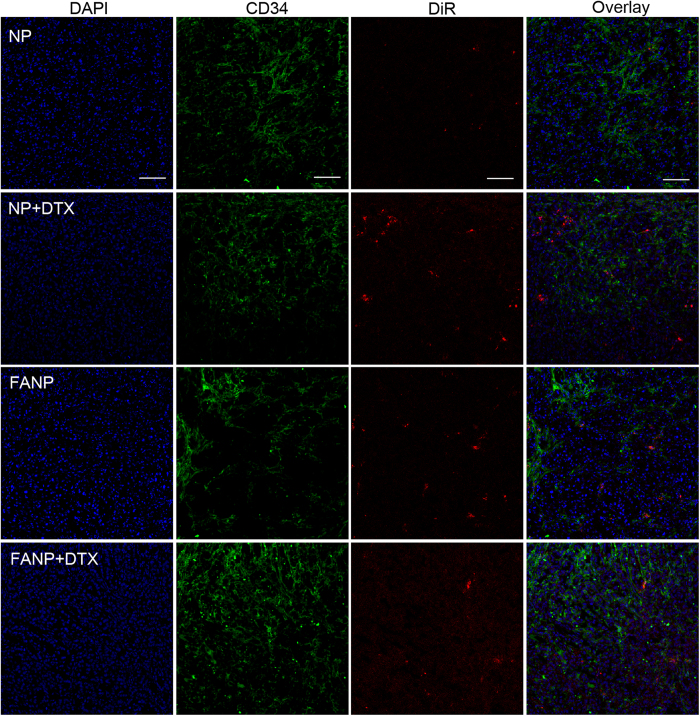
The accumulation of DiR labeled NP and FANP in tumor sections. Green represents CD34 antibody labeled blood vessels, red represents DiR labeled NP and FANP, blue represents DAPI labeled nucleus and bar represents 50 μm.

**Figure 7:**
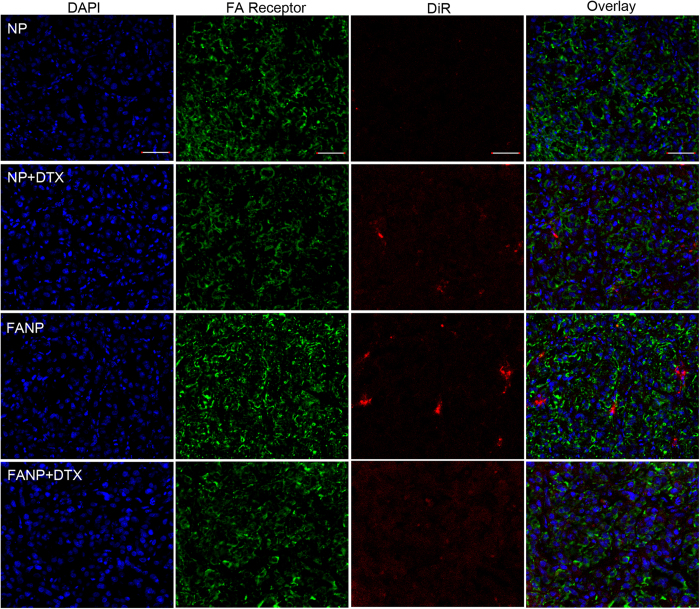
The accumulation of DiR labeled NP and FANP in tumor sections. Green represents FR antibody labeled FA receptor, red represents DiR labeled NP and FANP, blue represents DAPI labeled nucleus and bar represents 50 μm.

**Figure 8:**
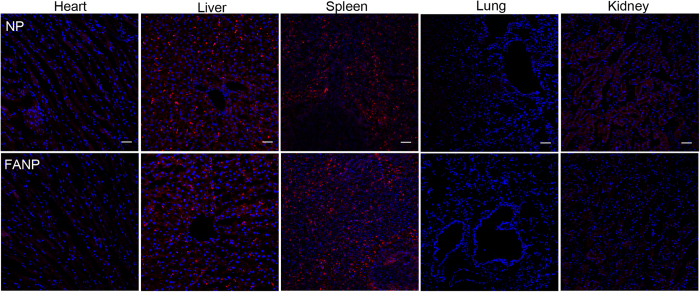
The distribution of DiR labeled NP and FANP in normal tissue sections. Red represents DiR labeled NP and FANP, blue represents DAPI labeled nucleus and bar represents 50 μm.

In order to investigate the distribution of FANP at the tumor sites in depth, the tumor sections were stained with CD34 antibody and FR antibody (Figs 6 and 7). DTX increased the accumulation of NP to a certain degree for the reason of G2/M retention effect. The fluorescence intensity of FANP was higher than non-targeted NP which largely attributed to the targeting effect of FA. Besides, the fluorescence signal of DTX pretreated FANP was the strongest of all, which mainly benefit from the outcome of DTX pretreatment and the target ability of FA compared with other groups. These results renewed the hope that utilizing the G2/M cell cycle to enhance the accumulation of targeted NP can improve the antitumor outcomes. What’s more, in normal tissue, NP and FANP mainly distributed in liver and spleen (Fig. 8), and modification of FA did not obviously alter the distribution in normal tissues, which was consistent with the *ex vivo* distribution.

## Discussion

Currently, cell cycle was rarely taken into consideration in the design of drug delivery system. However, many chemical drugs pointed at specific phase of cell cycle, for example, DTX acted on G2/M phase while methotrexate worked on S phase. But G0/G1, S, G2/M phase coexisted in cell cycle progression, consequently leading to modest therapeutic efficacy^35^,^36^. More importantly, the cellular uptake of NP has recently been emerged significant difference in different phase of cell cycle due to the absence of cell division^14^,^16^. In our experiment, the cellular uptake after various drug synchronization also demonstrated distinct difference, nocodazole (G2/M phase) > thymidine (S phase) > serum free (G0/G1 phase), which in agreement with previous research^14^. Additionally, the expression of receptor in different phases of cell cycle existed distinction. However, no investigation has been reported about the relationship between cell cycle and the accumulation of active tumor-targeting NP.

Herein, we designed a tumor targeting delivery strategy by combination with the cell cycle retention regent and ligand modified NP. The aim of our design is to enhance the targeting effect by application of the existing formulations in clinic. Since DTX is the most commonly used chemotherapeutic that could retain cells in G2/M phase and FA-targeted therapy has entered clinical trials for the treatment of cancer, we evaluated the combination effect of DTX and FANP. *In vitro*, the cellular uptake of NP or FANP was remarkably increased after pretreatment with DTX and displayed a positive correlation with DTX concentration. Our previous study showed that low dosage of DTX we used had no influence on cell apoptosis and arrested 90% cells in G2/M phase, proving that the effect of DTX on cell cycle progression lead to the increasing cellular uptake^18^. The results *in vivo* were also in consistent with that of *in vitro* experiments. Active tumor targeting FANP after pretreatment with DTX accumulated the most in tumor sites both *in vivo* imaging and in tumor slices.

In conclusion, in this study, we demonstrated combining active targeting nanomedicines with DTX pretreatment could indeed improve the tumor targeting delivery. Our study may provide a new and applicable direction for improving tumor treatment in clinic because the formulations used in this study were clinical available or under clinical evaluation.

## Methods

### Synthesis of FA-PEG-PCL

FA conjugated PEG-PCL (FA-PEG-PCL) was synthesized according to the scheme of Supplementary Fig. S8. FA was first activated with NHS according to previously reported literature^37^,^38^. Briefly, FA (0.88 g, 2 mmol), NHS (0.23 g, 2 mmol) and EDC (0.42 g, 2.2 mmol) were dissolved in anhydrous DMSO and stirred in the dark overnight. Then, tert-butyl N-(aminoethyl) carbamate (EDA-Boc) (0.39 g, 2 mmol) dissolved in pyridine was added into the mixture and reacted for another 24 h under the same condition^32^. Then (Tert-butyl N-(aminoethyl) carbamate) folic acid (FA-EDA-Boc) was obtained, and TFA (0.23 g, 2 mmol) was added to remove the Boc group. Finally the solution was precipitated by an excess amount of pyridine and obtained a yellow product (N-(2-aminoethy) folic acid) (FA-EDA). The product was dissolved in Dimethyl Sulfoxide-D6 and characterized by ^1^H-NMR spectra at 400 MHz using Varian Mercury400 (Varian Inc. USA). Furthermore, the obtained product were dissolved in methanol and tested on Triple Quad LC/MS (Agilent 6410, USA).

COOH-PEG-PCL (9 g, 0.5 mmol) was dissolved in a mixture of dichloromethane and DMSO in the presence of NHS (0.06 g, 0.5 mmol) and EDC (0.1 g, 0.6 mmol) and reacted overnight. FA-EDA (0.12 g, 0.5 mmol) dissolved in DMSO was added and stirred for another 24 h, then the resultant solution was dialyzed (MWCO 3000) using deionized water for 24 h. FA-PEG-PCL was obtained by lyophilization (yield: 86.5%).The obtained product was dissolved in CDCl_3_ and characterized by ^1^H-NMR spectra at 400 MHz using Varian Mercury400 (Varian Inc. USA).

### Preparation of FANP and NP

The FANP were prepared by an emulsion/solvent evaporation method^39^,^40^. In brief, 25 mg of MPEG-PCL, 2 mg of FA-PEG-PCL and 3 mg of FITC-PEG-PCL were dissolved in 1 mL of dichloromethane and then added to 5 mL of 0.6% sodium cholate hydrate solution. Subsequently, the solution was sonicated by a probe sonicator at 200 W for 150 s on ice. Then dichloromethane was removed by rotary evaporation and the FITC loaded FANP was condensed to a fixed concentration by ultrafiltration at 4000 g. To prepare DiR loaded FANP, 3 mg FITC-PEG-PCL was changed to 3 mg MPEG-PCL and 600 μg DiR was added, and then prepared as above. After changed FA-PEG-PCL to MPEG-PCL, the FITC loaded NP and DiR loaded NP could be obtained using above described procedures.

The mean particle sizes and zeta potentials of FANP and NP were determined by Malvern Zetasizer Nano ZS90 instrument (Malvern Instrument Ltd., UK). The morphology of FANP and NP was captured by transmission electron microscope (TEM) (JEM-100CX, JEOL, Japan).

### Cell cycle synchronization

To obtain A549 and U87 cells in different cell cycle, cells were treated with a serum-free culture medium for 48 h (G0/G1 phase), 2 mm thymidine (S phase) or 200 ng/mL nocodazole (G2/M phase) for 16 h at 37 °C in a humidified 5% CO_2_ atmosphere^41^,^42^,^43^,^44^. To assess the cell cycle distribution, all the cells above were collected and fixed in 70% ethanol overnight. After removal of ethanol, samples were washed three times with PBS, and then incubated with RNase A for 30 min. Next, samples were stained with PI and evaluated by a flow cytometer (Cytomics^TM^ FC 500, Beckman Coulter, Miami, FL, USA).

### Cellular uptake after cell cycle synchronization

A549 and U87 cells were pretreated with different cell cycle synchronization regent, then 0.1 mL of FANP or NP (200 μg/mL) were added into wells after the medium was removed. After incubated for 2 h, the cells were washed with cold PBS twice, trypsinized and responded in a proper volume of PBS. The cells were finally detected under a flow cytometer (Cytomics^TM^ FC 500, Beckman Coulter, Miami, FL, USA).

### Cellular uptake after pretreatment with DTX

A549 cells, U87 cells and L929 cells were seeded onto six-well plate at a density of 2 × 10^5^ cells per well. 0.1 and 0.5 μg/mL DTX was added into the plate and incubated for 24 h, then FANP and NP (200 μg/mL) were added into wells after the medium was removed. Two hours later, the cells were collected and detected by a flow cytometer. To evaluate the cellular uptake on A549 and L929 cells, cells were plated in six-well plates and cultured for 24 h, then 0.1 and 0.5 μg/mL DTX was added into the plate and incubated for 24 h, next CFPE-loaded LIP and FA-LIP were added into the plates at a same CFPE concentration. Then cells were washed, trypsinized and tested on a flow cytometer.

### *In vivo* imaging and tissue distribution

A549 xenografts bearing nude mice were prepared by subcutaneous injection of 1 × 10^7^ A549 cells suspended in 100 μL of PBS into male nude mice^45^. For the comparative analysis of the accumulation of nanoparticles in DTX pretreated and untreated tumor, A549 tumor were introduced into nude mice on both sides of flank. After the tumor volume reached about 100 mm^3^, the right tumors were injected with DTX at a dose of 2 mg/kg. The left tumors were injected with polyoxyethylene castor oil and ethanol mixed solution diluted by PBS instead. 24 h later, DiR loaded NP or FANP were injected via tail vein into A549 xenografts bearing mice at the DiR dose of 1.5 mg/kg. At 1, 2 and 4 h after administration, the mice were anesthetized and imagined by Live Cell Imaging System (LCIS, Maestro CRi, Inc., USA).

After that, all the mice were sacrificed and the main organs were harvested and imagined by Live Cell Imaging System. Normal organs and tumors were sectioned at a thickness of 16 μm. Firstly, the tumor slices were stained with rabbit anti-CD34 antibody (1:100) and goat anti-FR antibody (1:100) respectively overnight, followed by staining with Cy3-conjugated donkey anti-rabbit IgG and Cy3-conjugated donkey anti-goat IgG secondary antibody respectively. Then the slices were stained with 0.5 μg/mL of DAPI for 5 min at room temperature. Normal tissue slices were only stained by DAPI. After washing with PBS three times, the slices were immediately examined by a confocal microscope at corresponding excitation wavelength (LSM710, Carl Zeiss, Germany).

### Animals

Male nude mice (16–18 g) were purchased from experiment animal center of Sichuan University (Chengdu, China). All the animal experiments were performed in accordance with the principles of care and use of laboratory animals and were approved by the experiment animal administrative committee of Sichuan University.

### Statistical analysis

All the values were presented as mean ± SD. Statistical differences were evaluated with two-tailed Student’s t test. P value less than 0.05 were considered to be statistically significant difference.

## Additional Information

**How to cite this article**: Hu, G. *et al.* Utilizing G2/M retention effect to enhance tumor accumulation of active targeting nanoparticles. *Sci. Rep.* **6**, 27669; doi: 10.1038/srep27669 (2016).

## Supplementary Material

Supplementary Information
