# Supplementary material for: Utilizing G2/M retention effect to enhance tumor accumulation of active targeting nanoparticles
Source: Sci Rep. 2016 Jun 8;6:27669. doi: 10.1038/srep27669 (PMC4897711; doi:10.1038/srep27669)
Supplement: Supplementary Information [file srep27669-s1.pdf]

Utilizing G2/M retention effect to enhance tumor accumulation of active targeting nanoparticles

Guanlian Hu, Xingli Cun, Shaobo Ruan, Kairong Shi, Yang Wang, Qifang Kuang, Chuan Hu, Wei Xiao, Qin He, Huile Gao*

Key Laboratory of Drug Targeting and Drug Delivery Systems, West China School of Pharmacy, Sichuan University, No. 17, Block 3, Southern Renmin Road, Chengdu 610041, China.

*Corresponding author: gaohuile@scu.edu.cn.

Author Contributions: Guanlian Hu and Xingli Cun contributed equally to this work.

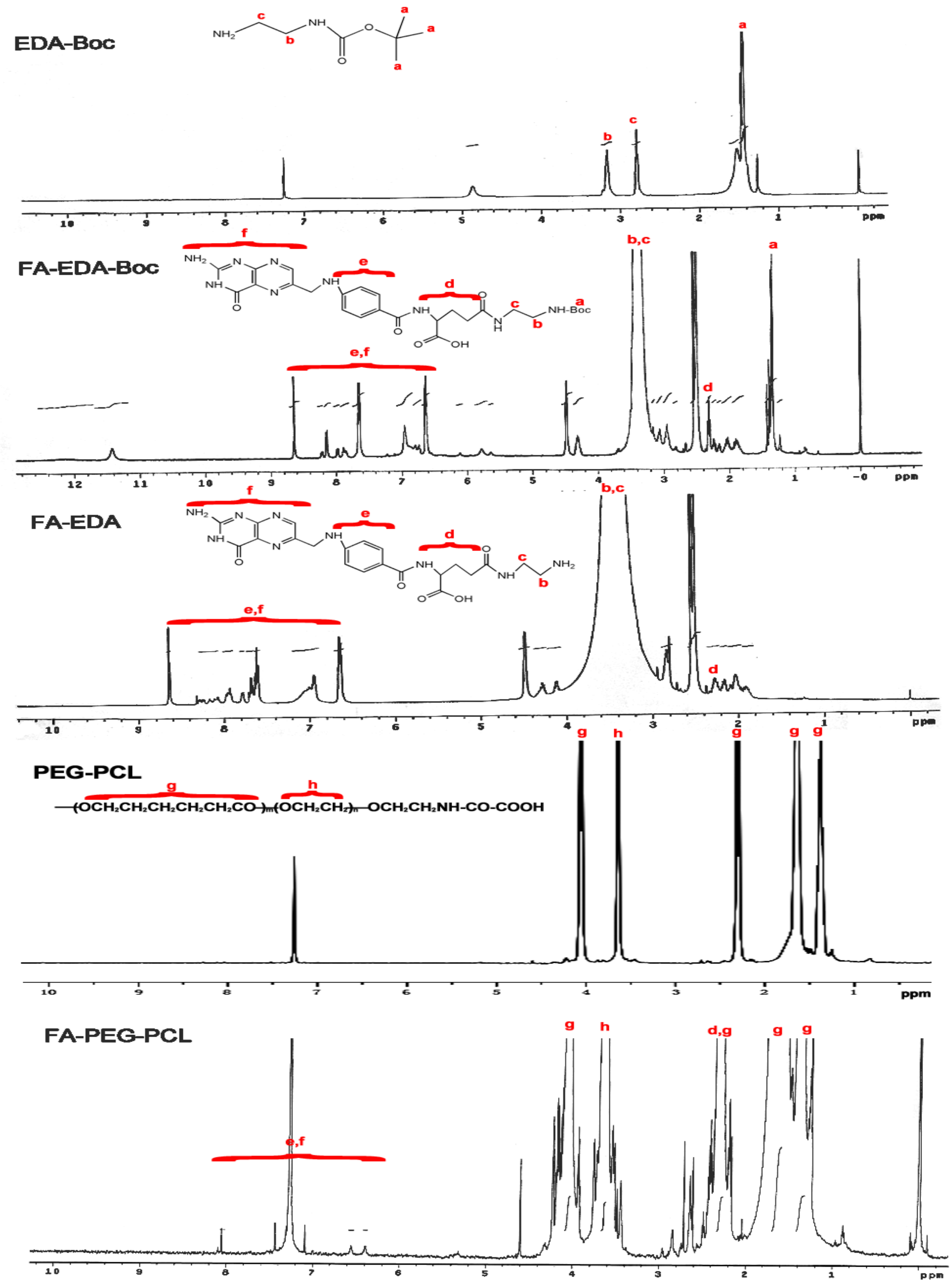

Supplementary Figure S1. ^{1}H-NMR spectra of EDA-Boc ($CDCl_3$), FA-EDA-Boc (Dimethyl Sulfoxide-D6), FA-EDA (Dimethyl Sulfoxide-D6), PEG-PCL ($CDCl_3$) and FA-PEG-PCL ($CDCl_3$), a, b, c, d, e, f, g, h represented the characteristic peak of each compound.

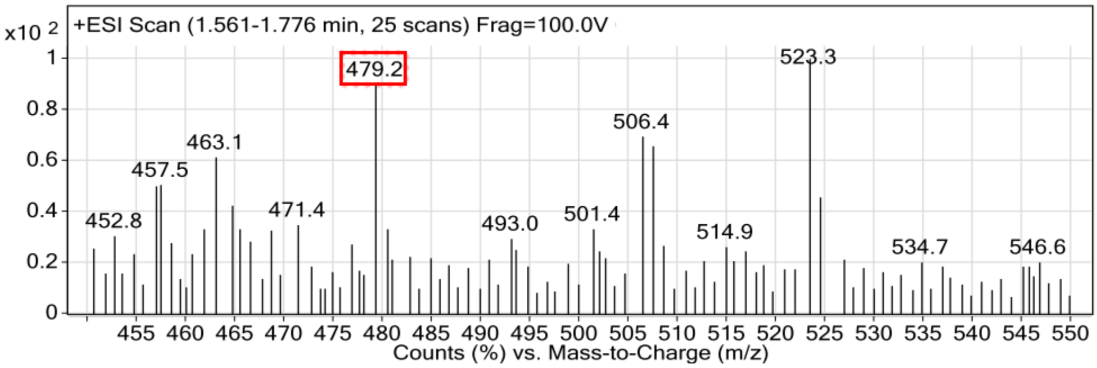

Supplementary Figure S2. Mass spectra of FA-EDA.

Supplementary Table S1. Particle sizes and zeta potentials of NP and FANP.

Formulation	Particle Size (nm)	PDI	Zeta potential (mv)
NP	91.45	0.136	-4.65
FANP	119.7	0.165	-4.82

Supplementary Table S2. Particle sizes and zeta potentials of LIP and FA-LIP.

Formulation	Particle Size (nm)	PDI	Zeta potential (mv)
LIP	103.0	0.191	-4.04
FA-LIP	101.7	0.216	-4.71

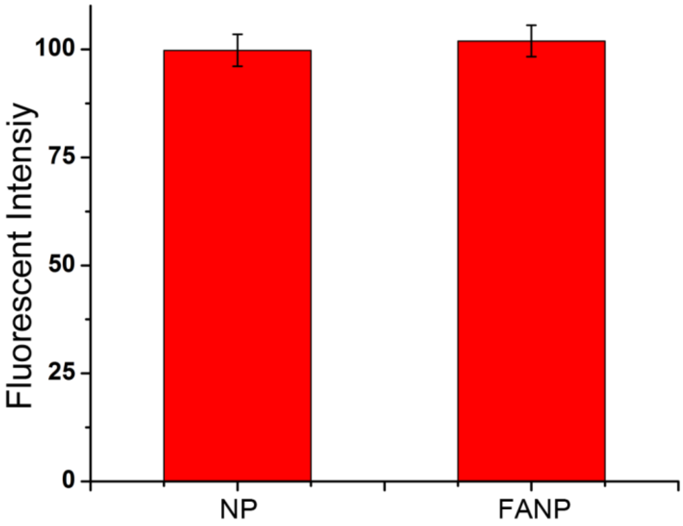

Supplementary Figure S3. The cellular uptake of NP and FANP on L929 cells 2 h after treatment with different NP.

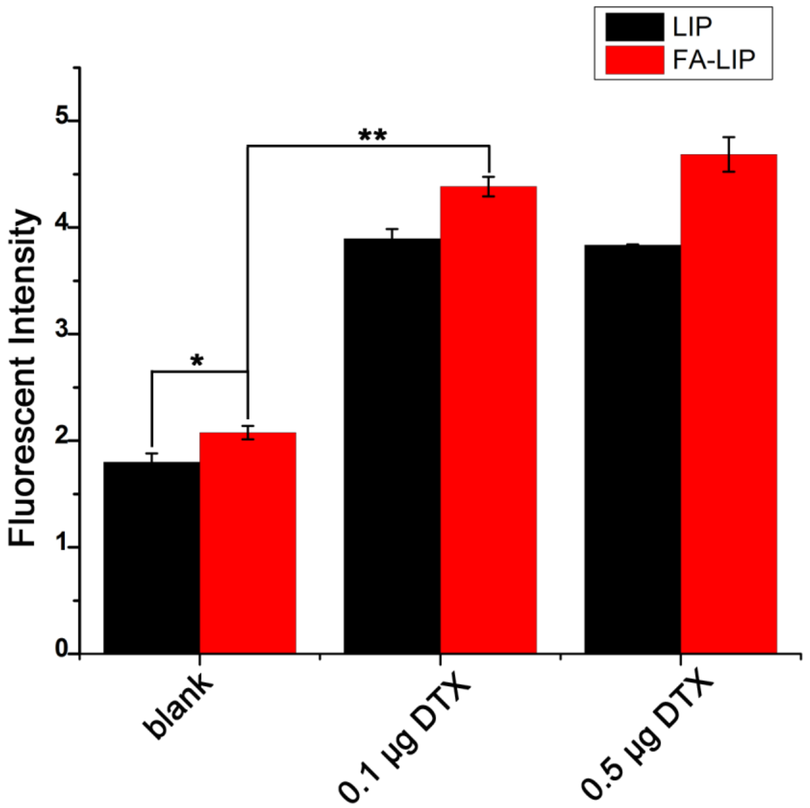

Supplementary Figure S4. The cellular uptake on A549 cells after pretreatment with 0.1 μg/mL or 0.5 μg/mL DTX for 24 h and then incubated with LIP or FA-LIP for 2 h *, ** and *** represent $P < 0.05$, $P < 0.01$ and $P < 0.001$ respectively between the marked groups.

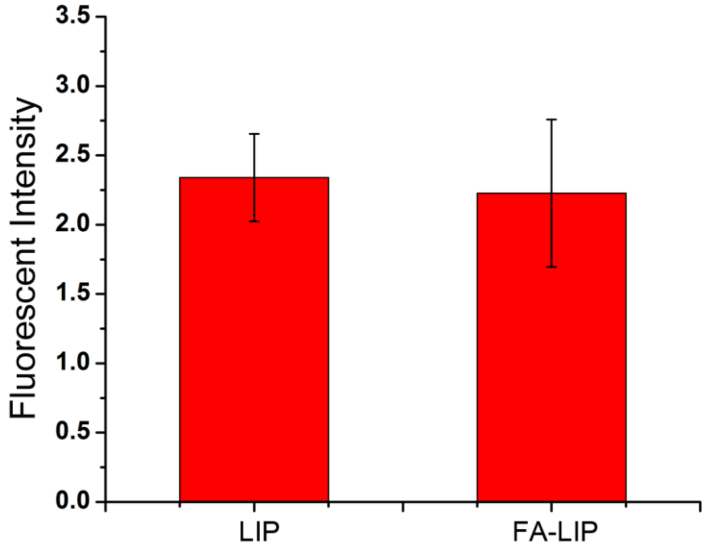

Supplementary Figure S5. The cellular uptake of LIP and FA-LIP on L929 cells 2 h after liposomal treatment.

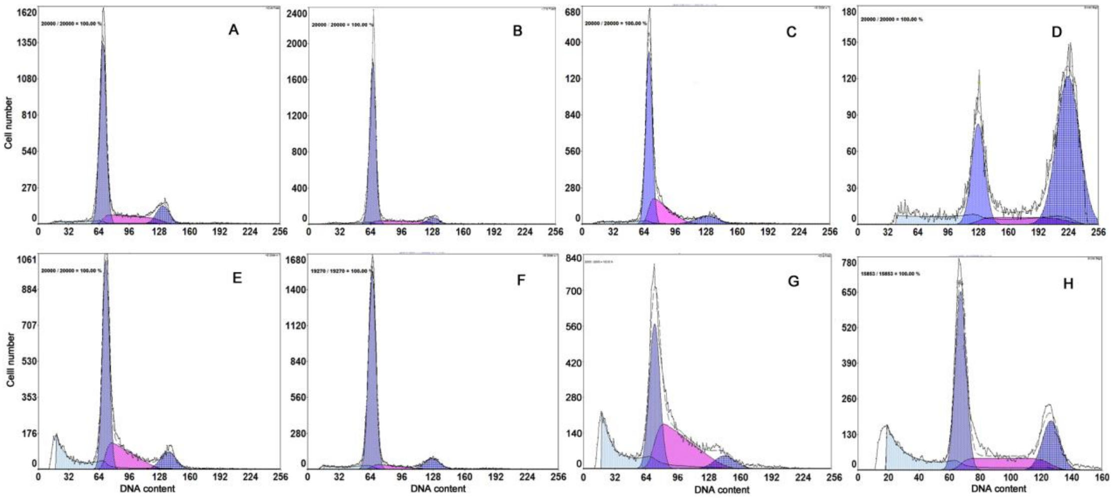

Supplementary Figure S6. The analysis image of cell cycle after drug-synchronized A549 cells (A, B, C and D) and U87 cells (E, F, G, H); (A and E) blank; (B and F) serum free, G0/G1 phase; (C and D) thymidine, S phase; (D and H) nocodazole, G2/M phase.

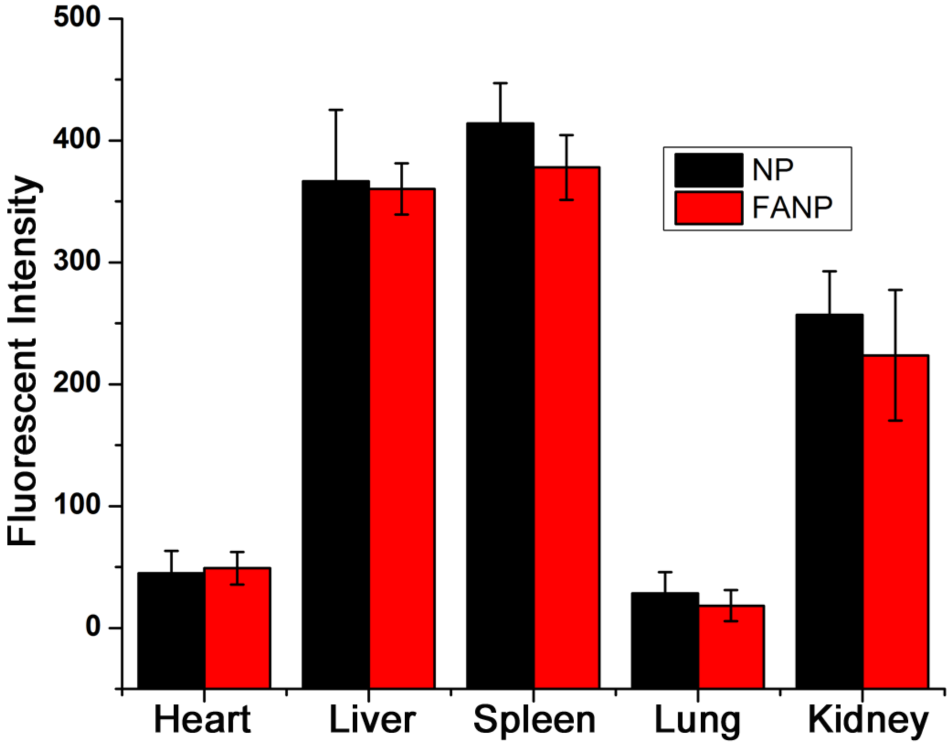

Supplementary Figure S7. Semi-quantitative analysis of fluorescent signals from normal tissues after 4 h injection with different particles.

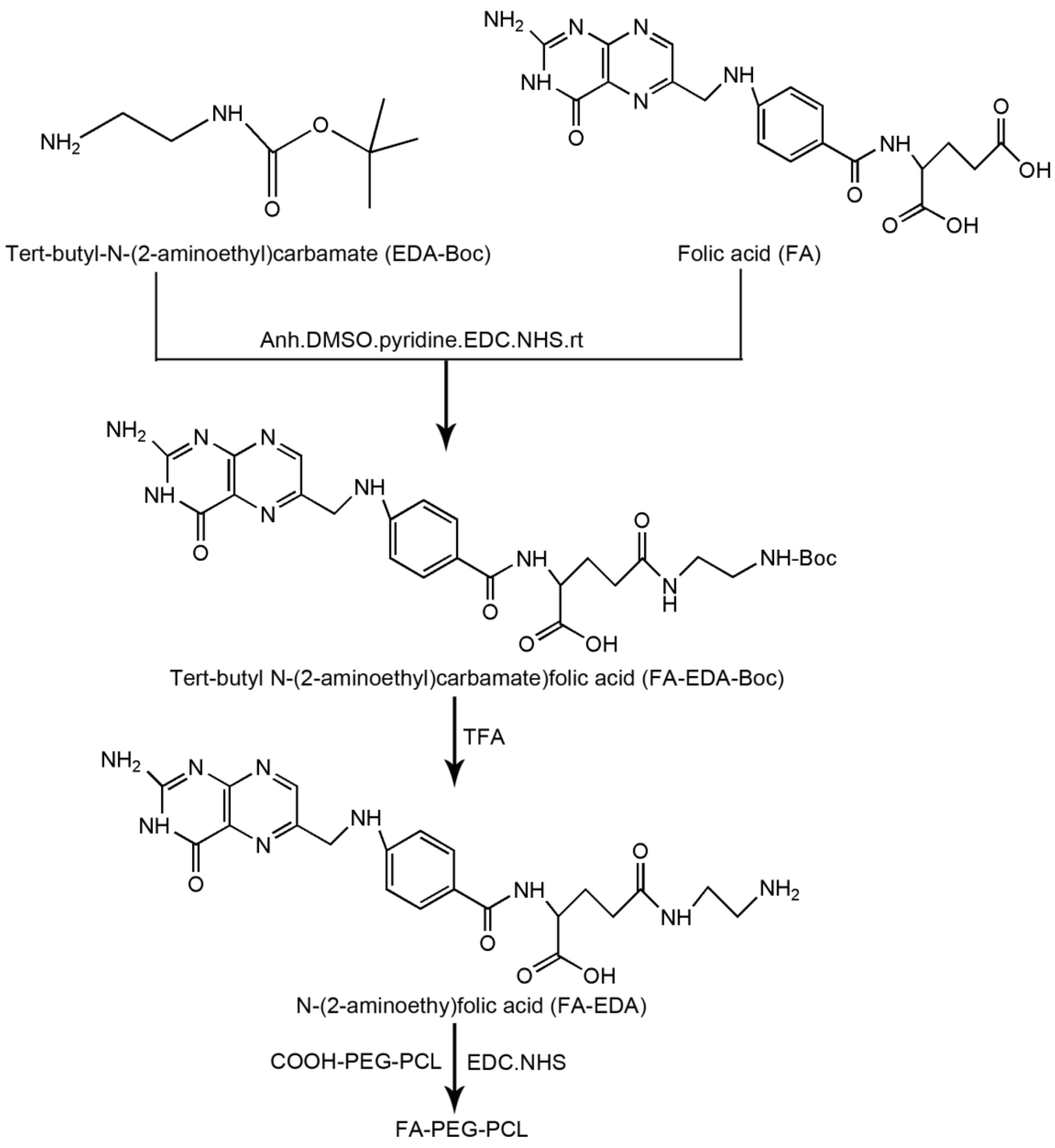

Supplementary Figure S8. Synthetic scheme of FA-PEG-PCL.

Supplementary Methods

Materials. DTX was purchased from Knowshine (Shanghai, China). Folic acid was purchased from Shanghai runjie chemicals (Shanghai, China). Methoxyl poly(ethyleneglycol-poly (ε-caprolactone) (MPEG-PCL) (Mw: 3k-15k), FITC-conjugated poly (ethyleneglycol)-poly(ε-caprolactone) (FITC-PEG-PCL) (Mw: 3 k-15 k) and carboxyl poly(ethyleneglycol)-poly(ε-caprolactone) (COOH-PEG-PCL) (Mw: 3 k-15 k) were synthesized as previously described [1]. 1-Ethyl-3-(3-dimethylaminopropyl) carbodiimide (EDC) and N-hydroxy-succinimide (NHS) were purchased from Chengdu Best Reagent Co. Ltd. (Chengdu,

China). Tert-butyl N-(2-aminoethyl) carbonate (Boc) and trifluoroacetic acid (TFA) were purchased from Hengli Chemicals (Chengdu, China). Thymidine and nocodazole were purchased from Sigma (St. Louis, Mo, USA). Soybean phospholipids (SPC) were purchased from Shanghai Taiwei Chemical Company (Shanghai, China). Cholesterol was bought from Chengdu Kelong Chemical Company (Chengdu, China). DSPE-PEG2000, DSPE-PEG2000-COOH and 1, 2-dioleoyl-snglycero-3-phosphoethanolamine-N-(carboxyfluorescein) (CFPE) were purchased from Shanghai Advanced Vehicle Technology L.T.D.Co.(Shanghai, China). Propidium iodide (PI) and RNAase were purchased from KeyGEN Biotech (Nanjing, China). DioctadecyL-3, 3, 3, 3-tetramethyl indotricarbocyanine iodide (DiR) were purchased from Biotium (Hayward, CA, USA). Rabbit anti-CD34 was purchased from Abcam (Hong Kong, China). Alexa Fluor 594-conjugated donkey anti-rabbit IgG was purchased from Jackson ImmunoResearch Laboratories, Inc (West Grove, PA, USA). FR-goat polyclonal primary antibody was purchased from Santa Cruz Biotechnology (CA, USA). Alexa Fluor 488 donkey anti-goat IgG (H+L) was purchased from Invitrogen (Carlsbad, California, USA). 40, 6-diamidino-2-pheylindole (DAPI) was purchased Beyotime Institute Biotechnology (Haimen, China). Plastic cell culture dishes and plates were purchased Wuxi NEST Biotechnology Co. (Wuxi, China). All other chemicals were analytical reagent grades and used without further purification.

Human lung adenocarcinoma cell line (A549) and human glioblastoma cell line (U87) were obtained from Shanghai Institute of Cell Biology (Shanghai, China).

Preparation and characterization of liposomes

CFPE-loaded liposome and FA-liposome were prepared by thin hydration method. PEGylated liposome (LIP) was prepared according to previous method [2]. Lipid compositions of the FA modified liposome (FA-LIP) were as follows: SPC/cholesterol/DSPE-PEG2000/DSPE-PEG2000-FA (molar ratio = 62:33:4:1). After all lipid materials were dissolved in chloroform, CFPE was added. Then the organic solvent was removed to obtain a thin lipid film. The hydration and ultrasound of liposomes was same as Liu *et al*'s study. Then the size and zeta potential of LIP and FA-LIP were detected by Malvern Zetasizer Nano ZS90.

References:

1. Gao H. *et al*. Precise glioma targeting of and penetration by aptamer and peptide dual-functioned nanoparticles. *Biomaterials*. **33**, 5115-5123 (2012).
2. Liu Y. *et al*. Paclitaxel loaded liposomes decorated with a multifunctional tandem peptide for glioma targeting. *Biomaterials*. **35**, 4835-4847 (2014).
